# Disappointments in Surgery

**Published:** 1900-12-29

**Authors:** 


### DISAPPOINTMENTS IN SURGERY.

We have heard so much in recent years about the perfection to which surgery has attained, and about the almost scientific precision with which the results of operative work can be foretold, that we need not be surprised to find that when failure occurs blame is apt to be attributed to the surgeon. To those within the circle, however, it hardly needs to be said that things are not quite so straightforward as those imagine who only know surgery by its pæans as raised on public occasions and in after-dinner speeches; and when Mr. D'Arcy Power, in addressing the Harveian Society last week, described some of the "Disappointments of Surgery," he was but giving form and shape to the experiences of many surgeons who have felt, although they may not have talked about, their own disappointments. It is not only in the great operations of surgery that results fail to come up to expectation. There are few operations more frequently performed than those of circumcision and for hare-lip, yet there are few which may occasionally give more unsatisfactory results. Then the recurrence of adenoids is a fertile cause of disappointment to parents. The operation for their removal may have been carried out with perfect success, but the predisposition remains. Adenoid tissue grows quickly, and the original condition is soon reproduced if reliance be placed too entirely upon operative measures. Too much therefore should not be promised as a result of removal.

Few operations perhaps lead to more disappointment than those carried out upon the kidneys. Curiously it is not always the patient by whom this is most felt, for sometimes, as when the operation is done for the relief of pain, the patient being relieved may be quite content, while the surgeon, seeing that other disastrous consequences have been set up, may be by no means so happy about what has been done. Among the troubles following abdominal operations one of the most annoying is the occurrence of fistulæ. There is no surgeon with any considerable experience in abdominal work who has not had occasion repeatedly to study their formation, even after laparotomies which at first promised success in every way. They may occur either immediately after the operation, when the escape of pus or other fluid has prevented the wound from healing, or at a period more or less remote after the wound has healed soundly. A fistula of the first kind can hardly be called a disappointment, as its occurrence is anticipated from the time the wound is left to granulate, but the more remote fistulæ are really disappointing. What, indeed, can be more vexatious than that a wound which has healed well and soundly should inflame after a week, a month, or even a year, at first without apparent cause, until at length a small abscess forms at or near the scar and a ligature presents? Although it may be true that the cause may be the same in every case, namely, an infected ligature, it seems not improbable that the general health of the patient may have something to do with the formation of an abscess in the more remote cases, for in the interval between the healing of the wound and the first sign of inflammation the patient appears quite well, and there is no evidence of any septic absorption going on. But the disappointment is the same whatever the cause.

Operations for hernia or for internal strangulations again are full of disappointments, sometimes possibly because it is impossible to fully diagnose the exact condition, sometimes because the operation has not been done soon enough, but sometimes because effectual as the operation may have been in rectifying the displacement which was the prime cause of the mischief, the patient dies from something beyond the art of surgery, a septicæmia, or a general infection which is beyond the reach of operation. As for the vermiform appendix, positive as are the results promised by some, and largely as the modern teaching of almost universal operation in such cases hinges on the certainty of success, Mr. D'Arcy Power warns us that these operations "afford so many surgical disappointments that I think it is never wise to promise the patient or his friends too much beforehand." A very proper caution. The great and immediate disappointment at the time of the operation is, he says, inability to remove the appendix, either because it cannot be found or because its attachments render it unsafe to complete the operation.

Another cause not only of disappointment but of great distress after various operations arises from the mental disturbances which sometimes follow their performance. These may show themselves in patients who have passed through life without any suspicion of mental unsoundness, although the event shows them to have been in a condition of unstable mental equilibrium. Much has been said of late years about the tendency of certain operations on the female genital organs to be followed by mental disorder, but Mr. D'Arcy Power gives instances which show that all sorts of operations may be followed by this unfortunate result. Perhaps the most disastrous, but at the same time some of the best-known surgical disappointments occur in connection with injuries to bones. Who is there who has not seen trouble after a Pott's fracture which has been well set shortly after the accident? The slight eversion and the accompanying flatness of the foot are always causes of annoyance to the patient. But even when there has been a seemingly good result, and time has been allowed to obtain firm union, the original deformity may recur if the patient be unusually heavy, or if the amount of repair has been miscalculated. Colles's fracture, again, is fruitful in disappointments, especially when it occurs in old people, while non-union of fracture in a long bone is more than disappointment, for when it occurs in children it may lead to the most disastrous results, even to amputation. Conical stumps, injuries to nerves, operations for the relief of chronic neuralgias, and the final results of many dislocations are all of them subjects from which it would be easy to find examples of disappointments in surgery.

Such was the sad confession of a general surgeon, but, as he pointed out, there is no reason to believe that the specialist is any better off. The ophthalmic surgeon has his troubles, the orthopædic surgeon is not always contented with his results, the gynæcologist too often finds himself landed in a sea of troubles after an apparently simple operation, whilst the general practitioner himself is soon disillusioned of the glamour of his profession, and is apt to become the most disappointed of any, for he sees the failure of all. Mr. D'Arcy Power draws the moral that "we should agree with Goethe that the most successful man is he who cares least for disappointments"; and a very good, comfortable moral too. Perhaps another also might be drawn, and that is that surgeons, in vulgar parlance, should not be "too cocky." A tendency has of late been far too much in evidence to regard a man as out of date who hesitates to regard the surgeon as the *deus ex machinâ* by whom all knots are to be unloosed. To read some of the contributions to American surgery makes one feel that operation for some conditions is so certainly indicated, and promises such results, that the physician who fails to urge its performance is either a knave, hoping for prolonged medical attendance on future attacks, or a fool ignorant of the modern aspects of surgery, and that the patient who shrinks from the knife is a mean-spirited cur. It is well, then, to be told by such an authority as Mr. D'Arcy Power that surgery has its disappointments as well as its triumphs.

